# Use of latent class analysis to identify multimorbidity patterns and associated factors in Korean adults aged 50 years and older

**DOI:** 10.1371/journal.pone.0216259

**Published:** 2019-11-13

**Authors:** Bomi Park, Hye Ah Lee, Hyesook Park

**Affiliations:** 1 Department of Preventive Medicine, College of Medicine, Ewha Womans University, Seoul, Korea; 2 National Cancer Control Institute, National Cancer Center, Goyang, Korea; 3 Clinical Trial Center, Mokdong Hospital, Ewha Womans University, Seoul, Korea; Indiana University Purdue University at Indianapolis, UNITED STATES

## Abstract

**Introduction:**

Multimorbidity associated with significant disease and economic burdens is common among the aged. We identified chronic disease multimorbidity patterns in Koreans 50 years of age or older, and explored whether such patterns were associated with particular sociodemographic factors and health-related quality-of-life.

**Methods:**

The multimorbidity patterns of 10 chronic diseases (hypertension, dyslipidemia, stroke, osteoarthritis, tuberculosis, asthma, allergic rhinitis, depression, diabetes mellitus, and thyroid disease) were identified via latent class analysis of data on 8,370 Korean adults aged 50+ years who participated in the sixth Korean National Health and Nutrition Examination Survey (2013–2015). The associations between multimorbidity patterns, and sociodemographic factors and health-related quality of life, were subjected to regression analysis.

**Results:**

Three patterns of multimorbidity were identified: 1) a relatively healthy group (60.4% of the population); 2) a ‘cardiometabolic conditions’ group (27.8%); and, 3) an ‘arthritis, asthma, allergic rhinitis, depression, and thyroid disease’ group (11.8%). The female (compared to male) gender was associated with an increased likelihood of membership of the *cardiometabolic conditions* group (odds ratio [OR] = 1.32, 95% confidence interval [CI] = 1.15–1.51) and (to a much greater extent) the *arthritis*, *asthma*, *allergy*, *depression*, *and thyroid disease* group (OR = 4.32, 95% CI = 3.30–5.66). Low socioeconomic status was associated with membership of the two multimorbidity classes. Membership of the *arthritis*, *asthma*, *allergy*, *depression*, *and thyroid disease* group was associated with a significantly poorer health-related quality-of-life than was membership of the other two groups.

**Conclusion:**

The co-occurrence of chronic diseases was not attributable to chance. Multimorbidity patterns were associated with sociodemographic factors and quality-of-life. Our results suggest that targeted, integrated public health and clinical strategies dealing with chronic diseases should be based on an understanding of multimorbidity patterns; this would improve the quality-of-life of vulnerable multimorbid adults.

## Introduction

Given aging populations, advances in medical care and public health policies, and improved living conditions, the co-occurrence of two or more chronic diseases in the same individual (multimorbidity) [1,2] is increasingly common [3–5]. A recent study of Koreans adults aged 50 and older found that more than one in four were multimorbid [6]. Multimorbidity is a public health concern, being associated with higher mortality, impaired functional status, a reduced quality-of-life, increased healthcare utilization, and a greater treatment burden [7–14]. The impact of multimorbidity can be much more complex (being synergistic) than the impact of individual diseases; health outcomes may differ by the disease combinations in play [3,15,16]. Thus, current single-disease-oriented public health strategies and clinical healthcare guidelines may be both incomplete and ineffective for multimorbid patients [17].

Certain chronic diseases tend to co-occur more often than expected by chance because they share pathophysiological pathways [18,19]. Identification of patterns of disease combinations and the characteristics of individuals exhibiting similar multimorbidity patterns may provide important information for policymakers and clinicians who seek to integrate strategic public health policy plans and healthcare management to address multimorbidity in at-risk groups more effectively. Even though multimorbidity has attracted increasing attention (because it is becoming the norm in the elderly; [17,20–25]), we still do not know how multiple chronic diseases cluster, the associated socioeconomic factors, and how multimorbidity affects quality-of-life.

Multimorbidity is highly complex. The use of a statistical approach to group a population into a limited number of subgroups with similar combinations of chronic diseases is much more practical than analysis of every possible disease combination. Therefore, we performed latent class analysis (LCA) based on the hypothesis that certain chronic diseases cluster. LCA identifies probabilistic rather than deterministic subgroups based on responses to a set of observed variables, and assumes that the pattern is explained by unobserved categorical latent variables of K classes [26,27]. Our objectives were: 1) to identify multimorbidity patterns in the general Korean population aged over 50 years using nationally representative survey data; and, 2) to explore whether such patterns were associated with certain sociodemographic characteristics and quality-of-life.

## Methods

### Data source and sample

We used data on adults 50 years of age and older who participated in the sixth Korean National Health and Nutrition Examination Survey (KNHANES) conducted in 2013–2015. KNHANES is a cross-sectional, nationally representative survey of the noninstitutionalized Korean population conducted by the Division of Chronic Disease Surveillance, Korea Centers for Disease Control and Prevention (KCDCP). KNHANES uses a stratified, multistage cluster sampling method based on geographical area, gender, and age. The details have been described previously [28].

### Measures

Chronic disease was based on self-reports, on whether participants had ever been physician-diagnosed with any disease on a pre-specified list. Analysis was limited to the 10 most common chronic diseases (hypertension, dyslipidemia, stroke, osteoarthritis, tuberculosis, asthma, allergic rhinitis, depression, diabetes mellitus, and thyroid disease; the prevalence of each is greater than 3% [29–31]) of the 28 diseases listed in the KNHANES. Multimorbidity was defined as the presence of two or more of these diseases in the same subject.

The sociodemographic variables evaluated included age, gender, household income, educational level, and occupation. The household income was the monthly income divided by the square root of household size [32], and was grouped into high and low using the median income as the cut-off. Educational level was categorized as low for those younger than 70 years who were high school graduates or less accomplished, and for those older than 70 years who were middle school graduates or less accomplished. In terms of occupational status, students and housewives were defined as unemployed; service workers. retailers, agriculture or fishery employees, technicians, mechanics, assemblers, and simple laborers were defined as manual workers; and managers, professionals, and office workers were considered to be non-manual workers. The health-related quality of life was assessed using the EuroQol 5 Dimensions (EQ-5D) instrument; this is a generic measure of health status. The EQ-5D features five dimensions (mobility, self-care, engagement in usual activities, pain/discomfort, and anxiety/depression); each dimension has three response options (1 = no problem, 2 = some problems, and 3 = a severe problem). The health-related quality of life was scored as a single value (the EQ-5D index score) using a validated algorithm [33,34].

### Statistical analysis

Disease prevalence, disease co-occurrence in the multimorbid, and the number of co-occurring diseases for each disease, were calculated. We used LCA to explore multimorbidity patterns. We examined one to six multimorbidity classes, and the optimal number of latent classes was determined based on the lowest Consistent Akaike Information Criterion (CAIC) and the adjusted Bayesian-Schwarz Information Criterion (adjusted BIC) [35–37], clinical significance, and interpretability [38]. After selection of an optimal model, each respondent was assigned to the class for which s/he had the highest computed membership probability. An average posterior probability greater than 70% indicates an optimal fit [39]. The characteristics of respondents in different latent classes were compared using the chi-squared test for categorical variables and ANOVA for continuous variables.

After identifying latent classes, as the second step of the analysis, multinomial logistic regression was performed to assess the association between each sociodemographic factor (age, gender, household income, educational level, and occupation) and latent class membership. Associations were assessed using odds ratios (ORs) with 95% confidence intervals (CIs). Each association was adjusted in terms of the other variables in the multivariate analysis. Subsequently, as the third step of the analysis, we investigated whether health-related quality of life varied by latent class membership by a simple analysis of variance. For these analyses, we used the BCH method to yield unbiased estimates of the class differences. This approach accounts for any uncertainty introduced by classification errors when estimating the model parameters of the latent classes [40,41]. The mean of the EQ-5D index score (with the 95% CI) was estimated for each latent class. The adjusted model considered gender, age, household income, educational level, and occupation. Bonferroni post-hoc testing was performed after all pairwise comparisons. As the EQ-5D index score is not normally distributed, it was log-transformed prior to analysis and then back-transformed. All tests were two-tailed, and a p-value <0.05 was regarded as statistically significant. All statistical analyses were performed with the aid of SAS software (version 9.4; SAS Institute, Cary, NC, USA). All estimates were subjected to sample weighting to reflect the complexity of the KNHANES sampling design.

### Ethics

The KNHANES was approved by the institutional review board of the Korea Centers for Disease Control and Prevention (approval nos. 2013-07CON-03-4C, 2013-12EXP-03-5C, and 2015-01-02-6C). Written informed consent was obtained from all participants.

## Results

A total of 8,370 participants aged over 50 years were included in analysis. The mean age was 62.5 years, and 46% were male. Of all respondents, 39% had two or more chronic diseases; the mean number of chronic diseases in multimorbid subjects was 2.6. Table 1 lists the prevalence and the proportions of multimorbidity, and the average number of comorbid diseases for each of the 10 chronic diseases included in the analysis. Hypertension (36.4%), dyslipidemia (22.1%), osteoarthritis (19.8%), and diabetes mellitus (14.4%) were the most prevalent diseases and at least one of the 10 diseases existed as a multimorbidity in over 60% of multimorbid patients; stroke occurred in 87.1% (the highest) and tuberculosis in 63.2% (the lowest). The number of co-occurring diseases varied between 2.2 and 2.9 depending on the index disease. Table 2 summarizes the LCA model fits. When up to six latent classes were considered, the smallest adjusted BIC (two-class model: 929.18; three-class: 797.28; four-class: 801.36) and CAIC (two-class model: 1016.91; three-class: 930.97; four-class: 981.01) were those of the three-class model; these classes were labelled *relatively healthy*, those with *cardiometabolic conditions*, and those with *arthritis*, *asthma*, *allergic rhinitis*, *depression*, *and thyroid disease* as revealed by the estimated probabilities of any particular chronic disease given membership of a latent class. Every respondent was assigned to one of the three classes based on the highest membership probability. The *relatively healthy* group included those with a low prevalence of all evaluated chronic conditions. The *cardiometabolic conditions* group was populated by those with high probabilities of hypertension, dyslipidemia, stroke, and diabetes mellitus. The latent class proportions were 60.4%, 27.8%, and 11.8% respectively (Table 3). The total proportion of classification error is 22% which is acceptable [39]. The mean age of the *cardiometabolic conditions* group was the highest (67 years). Both the *cardiometabolic conditions* group (40.2% male vs. 59.8% female) and the *arthritis*, *asthma*, *allergy*, *depression*, *and thyroid disease* group had higher proportions of females (17.7 vs. 82.3%); males constituted over 50% of the *relatively healthy* group (51.4 vs. 48.6%). In particular, most subjects were female in the *arthritis*, *asthma*, *allergy*, *depression*, *and thyroid disease* group (82.3%). Socioeconomic status was similar in the *cardiometabolic conditions* group and the *arthritis*, *asthma*, *allergy*, *depression*, *and thyroid disease* group; both groups evidenced higher proportions (compared to the overall figures) of individuals with lower household incomes, lower levels of education, and of unemployed status. The mean number of co-occurring chronic conditions was highest (2.9) in the *arthritis*, *asthma*, *allergy*, *depression*, *and thyroid disease* group (Table 4).

**Table 1:** The prevalence and characteristics of certain chronic diseases in 8,370 KNHANES 2013–2015 respondents aged 50 years and older.

	Prevalence	Cases with multimorbidity	Number of co-occurring diseases
Disease	% (SE)	%	Mean (SE)
Hypertension	36.4 (0.39)	69.4	2.2 (0.02)
Dyslipidemia	22.1 (0.26)	83.2	2.6 (0.03)
Stroke	4.1 (0.11)	87.1	2.9 (0.07)
Osteoarthritis	19.8 (0.26)	73.9	2.4 (0.03)
Tuberculosis	5.9 (0.14)	63.2	2.2 (0.06)
Asthma	3.6 (0.09)	83.5	2.8 (0.08)
Allergic rhinitis	8.1 (0.12)	67.6	2.3 (0.06)
Depression	5.6 (0.14)	74.7	2.6 (0.07)
Diabetes mellitus	14.4 (0.18)	84.4	2.7 (0.04)
Thyroid disease	3.9 (0.10)	74.9	2.5 (0.08)

KNHANES, Korea National Health and Nutrition Examination Survey; SE, Standard error

**Table 2:** A Comparison of the fit statistics of models featuring latent class analyses.

Number of latent classes	Likelihood ratio G^2^	Degrees of freedom	CAIC	Adjusted BIC
2	806.23	1002	1016.91	929.18
3	609.94	991	930.97	797.28
4	549.61	980	981.01	801.36

CAIC, Consistent Akaike Information Criterion; BIC, Bayesian Information Criterion

**Table 3:** Class membership and item response probabilities of the three latent classes.

	Latent Class		
Characteristics	Relatively healthy	Cardiometabolic conditions	Arthritis, asthma, allergy, depression, thyroid
Class membership probabilities (%)	60.4	27.8	11.8
Item response probabilities (%)			
Hypertension	0.18	0.75	0.42
Dyslipidemia	0.06	0.45	0.49
Stroke	0.01	0.12	0.02
Osteoarthritis	0.12	0.26	0.47
Tuberculosis	0.06	0.07	0.05
Asthma	0.02	0.04	0.14
Allergic rhinitis	0.07	0.04	0.24
Depression	0.03	0.04	0.21
Diabetes mellitus	0.04	0.39	0.13
Thyroid disease	0.02	0.03	0.14

**Table 4:** Characteristics of study respondents by latent class membership.

	Total	Latent Class			
Variable		Relatively healthy	Cardiometabolic conditions	Arthritis, asthma, allergy, depression, thyroid	*P*-value
	% (SE) or mean (95% CI)				
Age ^a^	62.5 (62.2–62.8)	60.8 (60.5–61.2)	67.0 (66.5–67.5)	63.4 (62.7–64.2)	<0.0001
Gender ^b^					
Male	46.3 (0.5)	51.4 (0.7)	40.2 (1.3)	17.7 (1.8)	<0.0001
Female	53.7 (0.5)	48.6 (0.7)	59.8 (1.3)	82.3 (1.8)	
Household income ^b^					
Low	52.4 (1.0)	47.4 (1.1)	65.8 (1.5)	57.0 (2.5)	<0.0001
High	47.6 (1.0)	52.6 (1.1)	34.2 (1.5)	43.0 (2.5)	
Education ^b^					
Low	80.9 (0.8)	78.7 (0.9)	85.2 (1.0)	87.1 (1.6)	<0.0001
High	19.1 (0.8)	21.3 (0.9)	14.8 (1.0)	12.9 (1.6)	
Occupation					
Unemployed	46.6 (0.8)	40.0 (0.9)	61.4 (1.3)	61.7 (2.2)	<0.0001
Unmanual	11.4 (0.5)	13.6 (0.7)	6.6 (0.7)	5.2 (1.0)	
Manual	42.0 (0.9)	46.4 (1.0)	32.0 (1.2)	33.1 (2.2)	
Number of co-occurring diseases ^a^	1.2 (1.2–1.3)	0.6 (0.6–0.6)	2.6 (2.6–2.7)	2.9 (2.8–3.0)	<0.0001

SE, standard error; CI, confidence interval

^a^ Values are presented as mean (95% CI)

^b^ Values are presented as % (SE)

A multinomial logistic regression analysis adjusted for age, gender, educational level, household income, and occupation showed that age increased the risk of being in one of the multimorbid groups—particularly the cardiometabolic conditions group—compared to the *relatively healthy* group. The risk of being in the *cardiometabolic conditions* group was higher for subjects in their 60s (OR = 3.08, 95% CI = 2.60–3.65), 70s (OR = 4.13, 95% CI = 3.44–4.95), and ≥ 80s (OR = 3.16, 95% CI = 2.39–4.19) compared to those in their 50s. The risk of being in the *arthritis*, *asthma*, *allergy*, *depression*, *and thyroid disease* group was higher for subjects in their 60s (OR = 1.98, 95% CI = 1.57–2.49) and 70s (OR = 1.54, 95% CI = 1.16–2.04) compared to those in their 50s, but no difference was found between subjects in their 50s and ≥ 80s. The risks of membership of the *cardiometabolic conditions* group (OR = 1.32, 95% CI = 1.15–1.51) and the *arthritis*, *asthma*, *allergy*, *depression*, *and thyroid disease* group (OR = 4.32, 95% CI = 3.30–5.66) were significantly higher for females than males. Lower household income (OR = 1.32, 95% CI = 1.14–1.52) and lower educational level (OR = 1.25, 95% CI = 1.05–1.49) significantly increased the risk of membership of the *cardiometabolic conditions* group. In addition, employed status decreased the risk of membership of both the *cardiometabolic conditions* group and the *arthritis*, *asthma*, *allergy*, *depression*, *and thyroid disease* group (Table 5).

**Table 5:** Sociodemographic factors of the latent classes as revealed by multinomial logistic regression.

	Latent Class		
Variable	Relatively healthy	Cardiometabolic conditions	Arthritis, asthma, allergy, depression, thyroid
	Odds Ratio (95% CI)		
**Unadjusted**			
Age (ref = 50s)			
60s	1	3.44 (2.94–4.03)	2.19 (1.76–2.72)
70s	1	5.36 (4.56–6.29)	2.04 (1.60–2.61)
80s	1	4.68 (3.66–5.99)	1.24 (0.73–2.09)
Gender (ref = Male)			
Female	1	1.57 (1.39–1.78)	4.90 (3.80–6.32)
Household Income (ref = High)			
Low	1	2.13 (1.88–2.42)	1.47 (1.21–1.80)
Education (ref = High)			
Low	1	1.55 (1.32–1.82)	1.83 (1.40–2.39)
Occupation (ref = Unemployed)			
Unmanual	1	0.32 (0.25–0.41)	0.25 (0.16–0.38)
Manual	1	0.45 (0.40–0.51)	0.46 (0.38–0.57)
**Adjusted**			
Age (ref = 50s)			
60s	1	3.08 (2.60–3.65)	1.98 (1.57–2.49)
70s	1	4.13 (3.44–4.95)	1.54 (1.16–2.04)
80s and older	1	3.16 (2.39–4.19)	0.82 (0.46–1.44)
Gender (ref = Male)			
Female	1	1.36 (1.19–1.55)	4.47 (3.41–5.85)
Household Income (ref = High)			
Low	1	1.33 (1.16–1.54)	1.10 (0.89–1.38)
Education (ref = High)			
Low	1	1.25 (1.05–1.49)	1.10 (0.83–1.46)
Occupation (ref = Unemployed)			
Unmanual	1	0.83 (0.63–1.09)	0.57 (0.37–0.87)
Manual	1	0.68 (0.59–0.79)	0.66 (0.53–0.82)

CI, confidence interval

Table 6 shows the association between class membership and health-related quality-of-life. When the means of the health-related quality-of-life of the latent classes were compared after adjustment for age, gender, household income, educational level, and occupation, individuals in the *arthritis*, *asthma*, *allergy*, *depression*, *and thyroid disease* group evidenced a significantly lower health-related quality-of-life (0.81) than the other two groups (0.97 for the *relatively healthy* group; 0.90 for the *cardiometabolic conditions* group) as revealed by Bonferroni post-hoc analysis for multiple comparisons.

**Table 6:** Associations between multimorbidity patterns and EQ-5D index scores^a^.

	Latent Class			*P*-value^b^
	Relatively healthy	Cardiometabolic conditions	Arthritis, asthma, allergy, depression, thyroid	
Unadjusted	0.95 (0.94, 0.96)	0.85 (0.83, 0.87)	0.77 (0.73, 0.81)	<0.0001
Adjusted^c^	0.97 (0.96, 0.98)	0.90 (0.88, 0.91)	0.81(0.80, 0.82)	<0.0001

EQ-5D, European Quality of Life 5 Dimension

Values are presented as geometric mean (95% confidence interval)

^a^Values are obtained from the BCH three-step approach

^b^*P*-value was calculated from Bonferroni post-hoc testing for multiple comparisons

^c^Age, gender, household income, education, and occupation are adjusted

## Discussion

We used LCA to identify three distinct multimorbidity patterns in the general Korean population: (1) a relatively healthy group; (2) a group with cardiocerebrovascular conditions including hypertension, dyslipidemia, stroke, and diabetes mellitus; and, (3) a group with arthritis, asthma, allergic rhinitis, depression, and thyroid disease. Our analysis not only indicated what other diseases were likely to co-occur in subjects with certain diseases but also allowed us to explore whether individuals with certain sociodemographic characteristics were more vulnerable to multimorbidity and the associated adverse health outcomes in terms of health-related quality of life.

It is difficult to directly compare our results to those of previous studies given the methodological differences in terms of study setting, disease spectrum and number, demographic factors, baseline health status of participants, and the statistical methods used [29], but the multimorbidity patterns we identified are in general agreement with those of prior works. Such similarities may indicate that chronic diseases aggregate because they share underlying risk factors (so one disease increases the risk of another) or that causalities are shared [19].

We found that hypertension, dyslipidemia, stroke, and diabetes mellitus were very likely to co-occur, as have several prior studies evaluating subjects with (predominantly) cardiocerebrovascular conditions [17,19,21,42–44]. In this study, the dyslipidemia response probabilities were very similar for the two multimorbid groups but dyslipidemia was classified as a *cardiometabolic condition* based on the clinical nature of the disease. It is known that hypertension, dyslipidemia, and diabetes mellitus are risk factors for cerebrovascular diseases, and may precede such diseases [45–48].

Although the mechanism behind the combination of arthritis, asthma, allergic rhinitis, depression, and thyroid disease remains unclear, similar groupings were evident in other studies. Arthritis and depression were co-grouped by Simoes et al. [19], asthma and allergy by Larsen et al. [49], and arthritis, asthma, and psychiatric symptoms formed a latent class in the work of Islam [17,44]. Thyroid function was strongly associated with neuropsychological function [50], the development or exacerbation of asthma [51,52], and progression of osteoarthritis [53].

We found that older age, being female, and lower socioeconomic status increased the risk of membership of the *cardiometabolic conditions* group and/or the *arthritis*, *asthma*, *allergy*, *depression*, *and thyroid disease* group, as compared to the relatively healthy group. Being in the older age groups increased the risk of being in the *cardiometabolic conditions* group three- to four-fold, but the pattern of arthritis, asthma, allergy, depression and thyroid disease was less dependent on age. This finding suggests that older people should be approached cautiously due to the possibility of multimorbidity with cardiometabolic conditions.

The *arthritis*, *asthma*, *allergy*, *depression*, *and thyroid disease* group were more strongly associated with a lower quality of life than the *cardiometabolic condition* group, although the quality of life was significantly lower in both multimorbidity groups compared to the relatively healthy group. Our previous study, based on participants 50 years and older from the same survey, found that the EQ-5D index score of patients with one of hypertension, diabetes mellitus, or dyslipidemia was 0.92, and that for patients with osteoarthritis only it was 0.83. These scores are similar to the EQ-5D index scores for the second and third patterns, respectively, in the present study. Therefore, the EQ-5D index score for the pattern reflects the impact of its constituent diseases on quality of life. The combination of diseases in the *arthritis*, *asthma*, *allergy*, *depression*, *and thyroid disease* group was strongly associated with more sensitive pain perception, physical limitation, functional impairment in daily activity, and quality of life in other works [19, 54], supporting our findings.

In addition, being female increased the risk of membership of the *arthritis*, *asthma*, *allergy*, *depression*, *and thyroid disease* group more than four-fold, and the female predominance in a musculoskeletal class and a headache-mental illness class was noted by Larsen et al. [49] as well. In addition, individuals assigned to this latent class had a lower quality-of-life. Therefore, females are significantly more likely to develop the disease cluster of arthritis, asthma, allergy, depression, and thyroid disease than males and so experience a lower quality of life for their remaining lifespan. Such gender inequality in healthy life expectancy caused by multimorbidity warrants public health interventions targeting the female elderly.

Multimorbidity is increasingly common; and current clinical practice guidelines, which focus on a single disease, are not responsive to the complex health care needs of multimorbidity. Our findings suggest the necessity of developing targeted prevention and treatment healthcare strategies that take the pattern of multiple chronic diseases into consideration. Given the cross-sectional association between multimorbidity pattern and health-related quality of life found in this study, it is possible that targeted efforts to prevent and manage the multimorbid patient will exert a positive effect on the health-related quality of life of patients with multiple diseases. In addition, given the existence of groups vulnerable to certain combinations of diseases, additional interventions are warranted in this vulnerable population.

Most previous studies on multimorbidity used a non-model approach such as counts of the most common disease combinations and their observed-to-expected ratios [55–57]; such methods are too simplistic. Recently, several studies have used statistical approaches such as cluster analysis [31,58,59], factor analysis [21,25,42,43], and LCA [17,22,36,44,60–62] to identify nonrandom multimorbidity clusters. We used LCA to identify subgroups based on structural equation modeling [63]. LCA is better than conventional clustering because LCA employs probability-based classification methods to choose an optimal number of classes based on various diagnostic tests [27,64]. This allowed us to group individuals into a limited number of latent classes and then analyze the differences between the classes.

Our study had certain limitations. Our study is based on limited number of chronic diseases with a prevalence of ≥ 3% recorded by the KNHANES. Previous studies that used latent class analysis also applied a prevalence cut-off for inclusion of 2–10% [17, 22, 30, 65, 66]. Other studies that applied different statistical techniques also established a minimal prevalence for the inclusion [29]. However, the pattern might have been different if greater number of diseases were included, so we additionally conducted an analysis that included 25 chronic diseases listed in the KNHANES. However, inclusion of diseases with a prevalence of < 3% did not change the class structure of the 10 originally included chronic diseases. Moreover, the response probabilities for the newly included diseases were too small to decide in which category each disease should be included. When we included a further six chronic diseases (all cancers combined, myocardial infarction, angina, rheumatic arthritis, renal failure, and atopic dermatitis) in addition to the 10 originally included chronic diseases in the LCA, the class structure was unchanged from the original pattern. Second, we only considered disease occurrence and, thus, not duration or severity. Finally, we cannot discuss causal relationships between diseases because the survey was cross-sectional in nature. However, using a large and nationally representative sample, we identified multimorbidity patterns, and associations between such patterns and both sociodemographic factors and health-related quality-of-life.

## Conclusion

This study demonstrates that there are distinct subgroups of patients with specific patterns of multimorbidity. Also, individuals of the three classes exhibited different sociodemographic characteristics and varied in terms of health-related quality of life. Our findings deepen our understanding of non-random associations between diseases; this will aid the design of timely, useful, effective, holistic healthcare and preventative strategies addressing the needs of multimorbid individuals and those at high risk of multimorbidity. In addition, given that the multimorbidity patterns are associated with poor quality-of-life and sociodemographic inequalities, targeted multimorbidity management is important to reduce the burden on the vulnerable population and to address the associated social inequalities.
